# Modelling the long-run learning impact of the Covid-19 learning shock: Actions to (more than) mitigate loss

**DOI:** 10.1016/j.ijedudev.2020.102326

**Published:** 2021-03

**Authors:** Michelle Kaffenberger

**Affiliations:** University of Oxford, Oxford, United Kingdom

**Keywords:** International education, Learning loss, Remediation, Covid-19

## Abstract

This paper uses a calibrated “pedagogical production function” model to estimate the potential long-term losses to children’s learning from the temporary shock of Covid-19 related school closures. It then models possible gains from two mitigation strategies. Without mitigation, children could lose more than a full year’s worth of learning from a three-month school closure because they will be behind the curriculum when they re-enter school and will fall further behind as time goes on. Remediation when children return to school reduces the long-term learning loss by half, but still leaves children more than half a year behind where they would have been with no shock. Remediation combined with long-term reorientation of curriculum to align with children’s learning levels fully mitigates the long-term learning loss due to the shock and surpasses the learning in the counterfactual of no shock by more than a full year’s worth of learning. Systems need to begin planning now for remediation programmes, and as they do so they should build programmes and train teachers in ways that can continue to produce benefits beyond the period immediately following reopening.

## Highlights

• Learning losses due to Covid-19 school closures could continue to accumulate even after children return.
• Simulations show a three-month school closure could reduce long term learning by a full year’s worth of learning.
• Short-term remediation when children return to school could reduce long term losses by half.
• Long-term system improvements could surpass pre-Covid learning trajectories by “building back better”.

## 1 Introduction

At its peak the Covid-19 pandemic forced more than 1.6 billion children temporarily out of school. While many education systems have attempted varying degrees of remote learning, it is widely accepted that the closures will produce substantial losses in learning (World Bank, 2020; Kuhfeld et al., 2020). A serious concern is that these short term learning losses could continue to accumulate after children return to school, resulting in large and permanent learning losses as many children who fall behind during school closures never catch up.

A recent study suggests that even temporary school closures can result in large medium-term lost learning. Andrabi et al. (2020) analyse the impact of the 2005 Pakistan earthquake on children’s learning four years later by comparing households that were close to the fault line with similar households that were farther away and not affected by the quake. Schools in the affected area were closed for an average of 14 weeks, a little more than 3 months. However, four years later children in the affected areas were not just three months behind, they were the learning equivalent of 1.5 years of schooling behind.^1^

The direct effect of the school closures alone cannot account for such large deficits in later test scores, suggesting affected children learned less each year after they returned to school because of the short-term interruption (Andrabi et al., 2020). One possible explanation is that the curriculum and instruction did not adapt to the children’s lower learning levels upon re-entry into school and hence the affected children fell further and further behind.

Leading education experts have called for adaptation of instruction when children return following the Covid-19 related closures. Rukmini Banerji, CEO of Pratham, the NGO in India which pioneered the “Teaching at the Right Level” approach, has said education systems should focus on “helping children catch up on basic foundational skills” when children return to school (World Bank Live, 2020). The World Bank has called for education systems to begin planning for large-scale remedial programmes (World Bank, 2020), and a consortium made up of UNESCO, UNICEF, the World Bank, the World Food Programme, and UNHCR has called for remediation to mitigate learning loss with a focus on literacy and numeracy for primary age children (UNESCO et al., 2020).

How much learning might be lost in the long run from the Covid-19 shock if nothing is done, especially if losses accumulate as the Pakistan study suggests they could? And how much of a difference might mitigation strategies make? This paper uses an existing pedagogical production function model (Kaffenberger and Pritchett, 2020), calibrated to replicate learning trajectories in low- and middle-income countries, to model the possible outcomes. To model learning loss, I introduce a learning loss shock for children currently in grade 3 and simulate how their learning is affected through grade 10.^2^

I find that if learning in grade 3 is reduced by one-third, roughly the amount of time many children are likely to be out of school, learning levels in grade 10 (compared to a counterfactual of the same children with no shock) are a full year lower. This is similar to the cumulative learning loss identified by Andrabi, Daniels, and Das (2020). Second, I find that if learning in grade 3 is reduced by half, which could reflect missing one third of a year of school plus additional learning regression while away from school (as in the phenomenon of “summer learning loss” (Slade et al., 2017)), then learning in grade 10 is 1.5 years lower than the counterfactual of no shock.

I then model two remediation approaches.

The first models short-term remediation efforts when these children return to grade 4. It assumes one-third of the grade 3 curriculum is covered during grade 4 before moving on to grade 4 topics. Starting in grade 5, instruction reverts to the previously established (pre-pandemic) curriculum and instructional levels. This is a “short-term” remediation model. Modelling this with the more conservative assumption of the loss of one-third of grade 3 learning from school closures, such short-term remediation mitigates about half of the grade 10 learning deficit, reducing the long-term impact of the shock to one-half of a school year.

The final scenario models an instruction reorientation strategy which combines short-term remediation with long-term adaptation of instruction to children’s learning levels. The steps that education systems will need to take to conduct remedial education, including instituting formative assessments to identify children’s learning levels, training and empowering teachers to conduct such assessments and adapt their instruction and pedagogical practices to students’ levels and needs, and prioritising children’s attainment of essential skills, are well-proven strategies for improving learning outside of a crisis context (Teaching at the Right Level (TaRL), 2018; Piper et al., 2018). The final model considers the outcomes if systems both conduct remedial instruction in grade 4 as described above and reorient instruction and practices to children’s learning levels on a long-term basis. This scenario not only fully mitigates the effect of the shock but increases grade 10 learning above the counterfactual of no shock by more than a full year’s worth of instruction.

Without the urgent and immediate attention of education systems to the question of how they will handle the learning losses from the temporary school closures, the consequences for today’s children will be long-run and large. Actions to protect children from these losses must be a top priority, even while the crisis persists.

## 2 Modelling learning

Modelling learning requires a specification of the parameters that drive the learning process. Changes to the parameters then allow modelling of counterfactual scenarios, such as shocks to the learning process or various policy priorities or scenarios. Kaffenberger and Pritchett (2020) develops a pedagogical production function (PPF) which models the learning gained by children at different points in a student distribution in a year of schooling. In this paper, I use this PPF to model the Covid-19 learning shock. This section provides a brief overview of the model, and more details can be found in Kaffenberger and Pritchett (2020).

The PPF is what, on average, child *i* with skill level *s* would learn if they attended grade *G*. In general terms this is represented as:

Learning process (LP) = LP^G^(s^i^)

Drawing on the findings of the emerging literature on learning profiles, Kaffenberger and Pritchett (2020) assumes a trapezoidal functional form for the PPF, as in the equation:

PPF(LP(w,h,r,πG),si)=hmin+rsi-(πG-w2) 0 if si<πG-w2if πG-w2< si < πG+w20 if si>πG+w2 

**Fig. 1:**
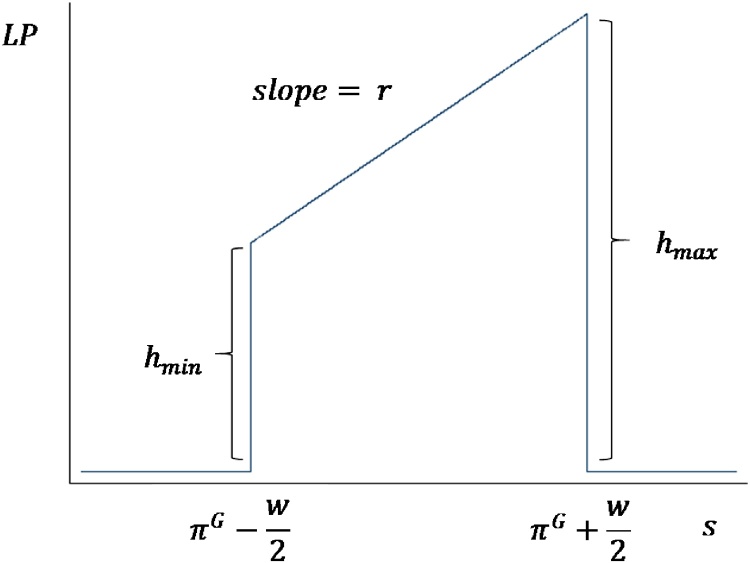
Modelling the learning process with a trapezoidal pedagogical production function.

Where the learning in grade *G* of student *i* of initial skill *s* is a function the width *w*, height *h*, slope *r*, and center $\pi^{G}$ of the trapezoid, as illustrated in Fig. 1.

Two distinct features follow from this functional form. First, the PPF assumes there is a range of initial skill levels within which children learn and above and below which they do not. If the instructional process is too advanced relative to student skill level (e.g. teaching division to children who cannot recognize numbers) or too rudimentary (e.g. teaching number recognition to students ready for geometry) no new skills are gained. The PPF or instructional process at grade *G* is centred on a specific skill level, $\pi^{G}$, and the width of the PPF, the range of initial child skills over which the instructional process produces learning, is the parameter *w*. Therefore a child too far behind ($s^{i}<\pi^{G}-\frac{w}{2}$) will learn nothing from attending grade *G* (Fig. 1).

Second, the trapezoidal shape has a slope parameter, *r*, so that learning can vary across the initial student distribution. Kaffenberger and Pritchett (2020) assumes an upward sloping trapezoid, with *r>*0, so that high performers learn more per year than low performers.^3^ *h_min_* is the amount learned by the child with the lowest initial skill level that learns anything at all, and *h_max_* is the amount learned by the child with the highest initial skill level that learns anything.

Fig. 1 illustrates this trapezoidal PPF learning process.

**Fig. 2:**
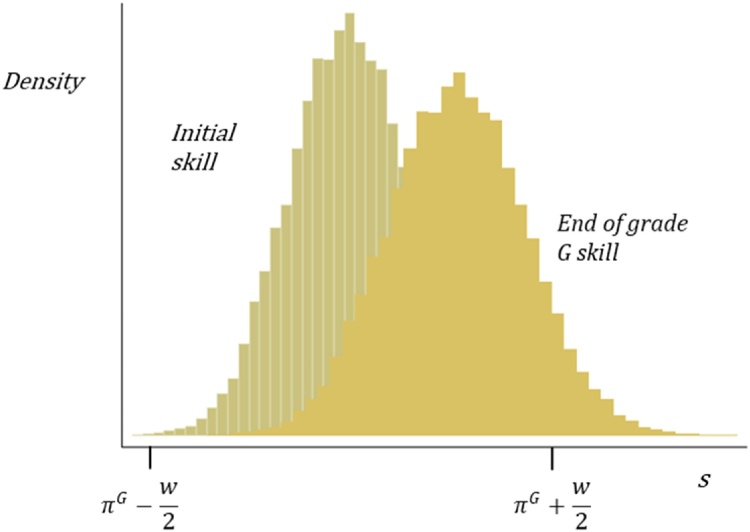
Initial and end of grade student skill distribution.

A final parameter, pace, *p*, represents the shift in the PPF from one grade to the next, as the level of instruction shifts to the next grade level. To model learning, the learning process is iterated by applying the PPF to an initial distribution of student skills to produce a new distribution based on the learning acquired in grade *G*. These dynamics are illustrated in Fig. 2. The PPF then shifts to the right according to the pace *p* to produce the learning for grade *G*+1.

Kaffenberger and Pritchett (2020) calibrate the model to replicate average grade 10 learning in mathematics in the seven low- and middle-income countries that participated in the PISA for Development (PISA-D) assessment.^4^ This is just one option for calibration and allows us to model long term learning (through at least grade 10).^5^ For the current modelling, this calibration allows us to model a “typical” learning process in a low- or middle-income country.^6^

PISA assesses children who are 15 years old and in school and in at least grade 7. Eligible 15-year-olds are on average in grade 10. In OECD countries, as a comparison, 89 percent of 15-year-olds are eligible, and PISA is standardized so that the mean score of these participating children is 500 and the standard deviation is 100. Among the PISA-D countries, 43 percent of 15-year-olds were eligible, the average score of participating children was 324, and the standard deviation was 74. In our calibration, we assume dropout is endogenous and that low performers dropout first. More details on the calibration process and dropout assumptions are provided in Kaffenberger and Pritchett (2020). We calibrate the PPF so that the top 43 percent of the grade 10 distribution roughly replicates the observed PISA-D results, with the combination of parameters that comes closest to replicating the PISA-D results given in Table 1. In Kaffenberger and Pritchett (2020) and in the following modelled scenarios, we use grade attainment data from the World Bank’s EdAttain database, averaged across the seven PISA-D countries, to model dropout after each grade. In this paper I assume enrolment and dropout stay constant at pre-pandemic levels, making the learning loss estimates optimistic if some children do not return to school (more details on this in Section III.C.).

**Table 1:** Calibrated parameters for reproducing average PISA-D scores.

Parameter	PISA-D calibrated parameters
W (width)	153
*h*_max_	49
h_min_	26
r (slope)	0.15
P (pace)	45
N(π^1,^ σ^1^)	N(020)

## 3 Modelling the Covid-19 learning shock and mitigation approaches

I use the calibrated PPF to model five scenarios. The first is the counterfactual of cohort learning with no shock, representing business-as-usual schooling. This serves as the comparison point for learning loss due to the shock – it is the counterfactual of no schooling disruption. Then I model two different learning loss scenarios, followed by two mitigation approaches.

### 3.1 How much long-term learning may be lost?

Using the calibrated PPF described in Section II, Kaffenberger and Pritchett (2020) estimates average grade 10 *cohort* learning among PISA-D countries. While the PISA-D assessment only provides information on the learning of the portion of the cohort that took the assessment, with the parameterized model it is possible to estimate learning trajectories and learning outcomes among the full cohort of in- and out-of-school children. This will serve as the base case counterfactual of learning with no shock.

The calibrated PPF produces average cohort learning at age 15 of 213 and cohort standard deviation of 126, on the PISA scale of OECD average 500 and standard deviation 100. This mean is much lower and the standard deviation much larger than those observed for the PISA-D eligible population, as cohort learning now includes a much longer left tail of low performers (i.e. those who never started or dropped out of school and were therefore ineligible for PISA-D). This distribution of cohort learning is the counterfactual to which the following modelled outcomes are compared.

To model Covid-19 related learning loss, I introduce a shock for the cohort of grade 3 students and model their learning trajectories and outcomes through grade 10.^7^ Today’s grade 3 students will be in grade 10 in 2027, three years before the SDG target completion date, making this a relevant cohort for understanding not only implications for long term learning loss but also repercussions for reaching international learning goals. I reduce their grade 3 learning gains by one-third, the equivalent of about a three-month school closure,^8^ and, in this initial scenario, assume no remedial efforts are made when children return but that schools return to “business as usual” curriculum and teaching. I also assume, for simplicity, (here and in the subsequent scenarios) no additional school dropout due to the closures so that dropout follows the same trajectory as in the counterfactual. Assuming no changes in dropout makes this a conservative estimate of learning loss, as discussed further in Section III.C.

**Fig. 3:**
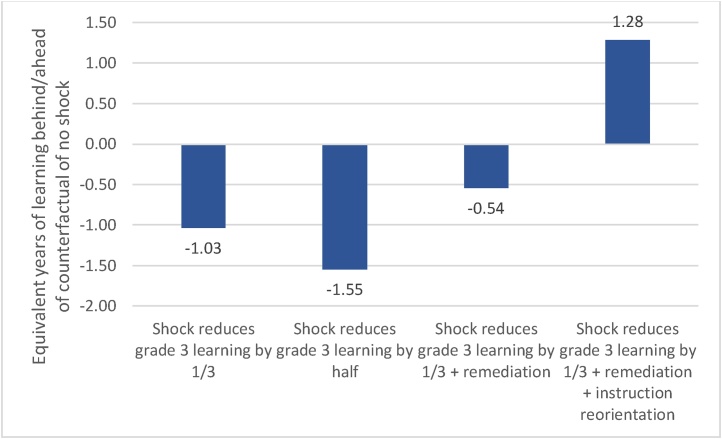
Modelling long-term lost learning from Covid-19 shock and mitigation strategies for the current grade 3 cohort: Equivalent years of learning behind/ahead in grade 10 compared to counterfactual of no shock.

**Fig. 4:**
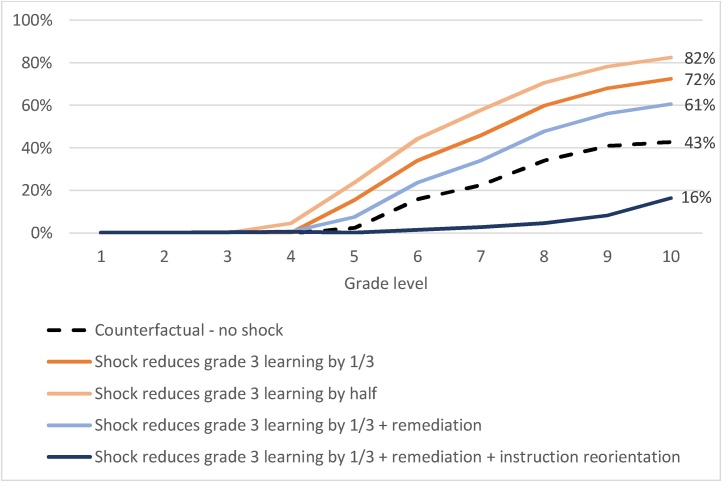
Percent of in-school children learning zero, due to falling behind the level of instruction, at each grade level. Covid-19 learning loss shock occurs in grade 3.

Similar to Andrabi, Daniels, and Das (2020), the simulation finds that reducing learning by one-third of a school year in grade 3 reduces later learning by a much larger amount. When this cohort of current grade 3 students reaches grade 10, their learning on average is a *full year lower* than what it would have been had there been no shock (Fig. 3).^9^ While this may at first seem extreme, the mechanism is clear. The lost learning puts children behind the curriculum, and without remediation they cannot keep up. They begin to fall outside the range of the PPF (i.e. outside the range of the curriculum and instruction) and cannot engage with the material. By grade 10, nearly three-quarters (72 percent) of children who are still in school have fallen outside the range of the PPF and hence are making no learning gains (Fig. 4).^10^

**Fig. 5:**
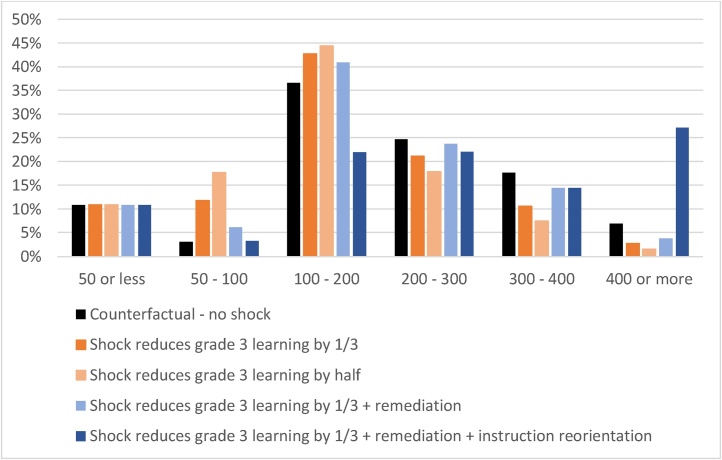
Percent of grade 10 cohort scoring in each band. Remediation + instruction reorientation produces learning gains far surpassing the counterfactual of no shock. On PISA-like scale; 400 roughly corresponds with the SDG goal of “minimum proficiency”. Note: The PISA scale is standardized to a mean among OECD countries of 500 and standard deviation of 100. The SDG goal of minimum proficiency roughly corresponds to a score of 400. Children scoring 50 or less either never started school or dropped out prior to grade 3 and so are unaffected by the shock and mitigation efforts.

Sustainable Development Goal 4 calls for all children to achieve minimum proficiency in reading and mathematics by 2030. One definition for minimum proficiency, established by UNESCO Institute for Statistics (UIS), is achieving a Level 2 on the PISA scale, roughly equivalent to a score of 400 (UNESCO, 2018). Because the model is calibrated to the PISA scale, it is possible to estimate the proportion of children who will achieve the SDG in each modelled scenario. The percent of the current grade 3 cohort that would reach the SDG goal of minimum proficiency in mathematics by grade 10 drops from 7 percent with no shock to just 3 percent reaching the SDG goal with the shock (Fig. 5).

I next model another variation of learning loss, reducing grade 3 gains by one-half. This could be thought of as the equivalent of direct learning loss from school closure and additional learning regression during the time out of school. “Summer learning loss” is an established phenomenon in high-income countries. A recent analysis suggests that in the United States, school closures due to Covid-19 could mean children return in the next school with less than 50 percent of the learning gains they would have had in math, and for some grades children could be a full year behind the gains they would have under normal circumstances (Kuhfeld and Tarasawa, 2020). Research on this topic is more sparse for lower income countries, but at least one study indicates that such “grade transition” loss does occur in lower income countries and can be severe (Slade et al., 2017). These suggest that even a 50 percent reduction in the grade 3 gains may be conservative in terms of the learning loss students experience due to Covid-19 closures.

In this scenario, the grade 10 learning deficit far surpasses the initial loss of one-half of a year’s learning. In grade 10, today’s grade 3 cohort has gained 1.5 years less learning than if the shock had not occurred. The percent of in-school children who have fallen outside the range of the PPF (i.e. behind the level of instruction) and are learning nothing is higher in every grade following the shock, reaching more than 80 percent in grade 10. Finally, this larger shock further reduces the percent of the cohort who reaches the SDG target for math to just 2 percent. The percent of the cohort with learning levels below 200, considered very low, rises to 73 percent.

### 3.2 How much difference could remedial efforts make?

There are steps that can be taken to mitigate some or all of these devastating outcomes. It has been widely acknowledged that remedial efforts will be needed when children return to school. A joint framework by UNESCO, UNICEF, the World Bank, the World Food Programme, and UNHCR has called on education systems to implement large scale remediation programmes (UNESCO et al., 2020). The World Bank has said that where full cohorts have missed content, especially in foundational subjects, “plans for teaching essential missed material should be integrated with plans for resuming progress through the curriculum,” (World Bank, 2020). How much could such remediation efforts mitigate the long-term effects of the learning shock?

For modelling mitigation, I use the more conservative learning loss assumptions from Section III.A., reducing grade 3 learning by one-third, and first model remediation when children re-enter school in grade 4. This assumes that education systems cover the material missed during school closures when children return to school. For example, if grade 3 children missed the last third of the school year, when they re-enter school in grade 4, they will cover the part of the grade 3 material they missed before moving on to the new grade 4 material. This is modelled by reducing the curricular pace from grade 3 to grade 4 by one-third, representing some of the grade 3 topics being covered in grade 4. After grade 4 the curriculum reverts to the previously established levels (and pace). This is the equivalent of a short-term remediation effort.

Short-term remediation makes up for some of the long-term learning loss. Average grade 10 cohort learning with remediation is half of a school year higher than without remediation. It does not, however, fully make up for the learning loss of the shock. This is because instructional time required for remediation reduces the time available for the regular grade level instruction, so learning losses are partially but not fully compensated. Cohort learning in grade 10 is still 0.55 years behind the counterfactual of no shock (Fig. 3). In the remediation scenario the percent of the grade 10 cohort who reach the SDG for mathematics is 4 percent, compared with 3 percent with the shock and no remediation, and 7 percent in the counterfactual with no shock (Fig. 5).

Finally, I model a scenario of remediation plus longer term “reorientation” of instruction. This scenario acknowledges the opportunity that education systems have to “build back better,” and particularly to do so building on capabilities they may gain in implementing remediation programmes. Evidence suggests that curricula, and resulting instruction, in many developing (and developed) countries are overambitious, covering many topics with limited time allocated to each. Teachers under pressure to complete the curriculum must rush through the content before students can fully grasp the new knowledge. As a result, many children miss out on foundational and essential skills and fall further and further behind (Beatty and Pritchett, 2015; Glewwe et al., 2009). This is represented in the model as children fall outside the range of the PPF, unable to keep up with the pace of instruction, and stop making learning gains.

In Uganda, for example, a study of the national curriculum found that foundational English competencies receive very little emphasis before children are expected to move on to higher order skills (Atuhurra and Alinda, 2017). In 2015 Tanzania reformed its grade 1 and 2 curricula, which at the time consisted of eight subjects including Vocational Skills and Information and Communication Technology. The reform radically simplified the curriculum, placing 80 percent of instructional time on foundational literacy and numeracy, and preliminary evidence shows large gains in the foundational subjects as a result (Mbiti and Rodriguez-Segura, 2020). These studies, and others, suggest that reorienting curriculum to children’s ability levels and ensuring adequate coverage of topics so that children can gain competency can substantially improve learning. The “Teaching at the Right Level” approach, pioneered by Pratham, provides further evidence that such tailoring of instruction to children’s ability levels can have large impacts on children’s learning, and such programmes are now expanding and scaling throughout Africa (Teaching at the Right Level (TaRL), 2018).

If systems implement effective remediation when schools reopen, many of the building blocks for such adaptations of instruction to meet children where they are would be put in place. For a system to implement remediation efforts effectively, as modelled in the first mitigation scenario, teachers and schools require some ability to conduct formative assessments, to determine children’s learning levels when they return, and to identify the subjects in need of remedial attention. It also requires the ability to adapt instruction to accommodate these needs, tailor content, and adjust curriculum. Doing so will require that teachers receive training and professional development and that they are allowed and empowered to adapt the curriculum they typically are required to fully cover.

If systems embrace the current crisis as an opportunity to “build back better”, they could build on these remediation efforts to maintain good practices in the long run. This could include ongoing use of formative assessments as a part of standard instructional practices, adjustment (or reform) of curriculum to better match the level and pace of children’s learning, and ongoing support, such as through coaching or structured pedagogy (Piper et al., 2018), to help teachers put new practices and curriculum into practice. Such efforts will look different in different systems, and the exact form would need to take the context and the system’s existing capabilities and constraints into account.

In such a scenario, in which an education system not only conducts remediation immediately upon return to school, but also carries the capabilities it gains in formative assessment, adaptation of instruction, and ensuring all children master foundational skills into the future, what could be the result for long-term learning? In this final scenario I build on the first mitigation scenario, modelling the shock that reduces grade 3 learning gains by one-third plus remediation as described above. In addition, I reorient the curriculum for the remaining school years through grade 10 and assume that after grade 4 the pace is “optimised” to children’s learning levels. The “optimised” curricular pace draws from Kaffenberger and Pritchett (2020) which, for the calibrated PPF model, identifies the curricular pace that maximizes grade 10 learning outcomes. This optimised pace is a pace of 35 on the scale used for the model, a reduction of 22 percent from a pace of 45 which reproduces PISA-D learning outcomes (and is “overambitious” per children’s learning levels). This implies better alignment between instruction and children’s learning levels and paces in each grade. By reducing the pace, fewer children fall behind the level of instruction because sufficient time is spent on content before moving on. With the slower pace, more children stay in the range of the PPF (and continue learning) longer, increasing learning outcomes.

This scenario that combines short-term remediation with long-term reorientation of instruction to children’s learning levels not only fully mitigates the long-term learning loss due to the shock, but also surpasses the learning in the counterfactual of no shock by more than a full year’s worth of learning. Remediation combined with long term reorientation of instruction produces average cohort learning of 259 in grade 10 on the PISA-like scale, a whopping 2.3 years’ more learning than if the shock had gone unmitigated (Figure 3). This is also 1.3 years’ more learning than the counterfactual of no shock occurring. With remediation and instruction reorientation, 27 percent of the cohort achieves the SDG—nine times more than had the shock gone unmitigated, and even nearly four times more than the counterfactual of no shock (Figure 5).

This scenario achieves such large learning gains because the reorientation of instruction enables more children to continue learning for longer. The percentage of in-school grade 10 children who are learning zero (because they have fallen below the range of instruction) in the remediation plus reorientation scenario is just 16 percent, compared with 72 percent with the unmitigated shock, and 43 percent in the counterfactual of no shock (Fig. 4). Because instruction moves at a pace with which children can keep up, they continue learning and gaining new competencies.

Much of the gains come from reducing the number of children scoring between 100 and 200 on the PISA-like scale, and greatly increasing the percent scoring above 400 (Fig. 5).

The results from all four scenarios and the counterfactual of no shock are summarized in Table 2. It is clear that across all measures, the remediation plus instruction reorientation scenario performs better even than the counterfactaul of no shock, truly representing a "build back better" scenario.

**Table 2:** Summary of modelled outcomes of Covid-19 learning loss shocks and mitigation scenarios.

	Grade 10 average cohort learning (PISA-like scale)	Equivalent years of learning ahead/behind of counterfactual in grade 10	Percent of in-school children in grade 10 learning zero	Percent of grade 10 cohort above PISA 400 (achieving minimum proficiency)	Percent of grade 10 cohort below PISA 200 (very low learners)
Counterfactual – no shock	213	N/A	43 %	7%	51 %
Shock reduces grade 3 learning by 1/3	175	−1.04	72%	3%	65 %
Shock reduces grade 3 learning by 1/2	157	−1.55	82%	2%	73 %
Shock reduces grade 3 learning by 1/3 + remediation	193	−0.55	61%	4%	58 %
Shock reduces grade 3 learning by 1/3 + remediation + instruction reorientation	259	1.27	16%	27 %	36 %

On one hand, it may be surprising that relatively simple efforts to tailor instruction to children’s ability levels could produce such large learning gains. These results, however, are in line with the large impacts achieved by many programmes that have worked to reorient instruction to children’s ability levels. Pratham’s TaRL approaches, for example, have effect sizes ranging from 0.08 to 0.70 standard deviations for relatively short-term programmes (ranging from 10-day remedial camps to a full year of reoriented instruction for foundational subjects) (TaRL, 2018). A computer-aided learning programme that adapted instruction to children’s individual learning levels achieved improvements of 0.29 standard deviations over just a 4.5 month period in India (Muralidharan et al., 2019). The Tusome programme in Kenya achieved impacts of 0.6–1.0 standard deviations in English and Kiswahili learning outcomes after one year through a multifaceted programme that reoriented literacy instruction to ensure all children were learning (Freudenberger and Davis, 2017; Piper et al., 2018; Wilichowski et al., 2020). It is entirely conceivable that adjusting curriculum pacing and instructional focus to be in line with children’s pace of learning could produce such large long-term effects.

On the other hand, perhaps it is not surprising at all that a multifaceted effort to conduct remediation combined with long-term reorientation of instruction would produce such large gains. Here, it is worth keeping in mind that the large, modelled impacts have incorporated a major shock that, if left unmitigated, would have reduced long term learning by a full school year. An effort that can so fully mitigate a major shock to learning and far surpass the counterfactual learning if there had been no shock deserves attention and consideration by education systems planning for reopening.

### 3.3 Limitations to the modelled learning loss

The modelling of learning losses in the above scenarios has limitations. The simulations assume no additional dropout as a result of school closures, rather assuming that enrolment and completion rates maintain pre-shock levels once schools reopen. This suggests that the modelled learning loss may be optimistic, and actual learning losses could be even worse if many children do not return to school. After being out of school for an extended time, some (or many) children may not return (UNESCO et al., 2020). During their time out of school, some children may be put to work to help support their household and have to remain in employment once schools reopen. Reductions in children returning to school would further reduce learning outcomes (increasing learning loss) due to the shock.

This model also does not build in macro shocks such as reductions in education spending or losses of parental income. The World Bank forecasts that education budgets in 2020 could fall by as much as 4.2 percent in Sub-Saharan Africa and 6.4 percent in South Asia, though there is much uncertainty in making such forecasts at this stage in the crisis (Al-Samarrai, 2020). The same World Bank report states that in low-income countries households contribute, on average, 29 percent of education funding, and households are being hit hard economically in the crisis. Reduced income will reduce households’ abilities to invest in education. Remittances are also expected to drop significantly, and education is often among the top uses of remittances by receiving households. While education spending is poorly correlated with learning outcomes (World Bank, 2018; Beatty et al., 2018; de Ree et al., 2018), large reductions, if maintained in the long run, could have detrimental effects on outcomes. Thus, the scenarios simulated in this paper may be even more optimistic if economic constraints further reduce schooling attainment and quality of instruction for those in school.

Finally, the model does not currently allow differentiation across countries. It is calibrated to the average learning across the countries participating in the PISA-D assessments. Future work aims to develop country-specific calibrations, but doing so is beyond the scope of the current paper.

## 4 Conclusion

The Covid-19 pandemic, which began as a health crisis, has also had tragic economic and educational consequences. The model presented in this paper suggests that the long-term repercussions for children’s learning could be devastating, with today’s grade 3 students losing as much as 1.5 years’ worth of learning (or more) by the time they reach grade 10 as a consequence of their time out of school. Governments can, however, introduce measures that mitigate some or all of these consequences. The model suggests that effective remediation efforts immediately upon return to school could reduce long-term learning loss for the cohort of grade 3 students by half. Beyond immediate efforts, there is an opportunity for systems to use the skills gained from implementing large-scale remediation programmes to reorient instruction to better match children’s skill levels in the long run. Such efforts, the model suggests, could not only fully mitigate the consequences of the shock but also surpass learning outcomes compared to the counterfactual of no shock.

All of these mitigation efforts require planning. As systems continue their remote learning programmes, they will also need to begin planning for reopening, putting in place the tools for remedial programmes and, if feasible, beginning to train teachers remotely. As they do so, they should consider how they can build programmes and train teachers in ways that can continue to produce benefits beyond the period immediately following reopening. The present crisis presents an opportunity for systems to build back better.

## Footnotes

1 Their test scores were 0.24 standard deviations behind unaffected children and the average annual gain is only .15. The study also finds that, due to government and international relief efforts, affected households were no worse off economically than unaffected households (in terms of wealth, consumption, or infrastructure), so the learning affects are not consequences of economic hardships.

2 The choice of grade 3 is illustrative, as some learning has already occurred, but enough years remain to model long-term learning loss. The shock could be modelled for children in any grade.

3 This is based on the literature on learning profiles showing that low performers often learn less per year than high performers. It could just as easily be reversed, such as in a remedial environment, and modelled so that low performers learn more and high performers learn less.

4 The PISA-D programme adapted the traditional PISA assessment to lower income countries and included capacity building for participating countries to conduct the assessment. Participating countries included Cambodia, Ecuador, Guatemala, Honduras, Paraguay, Senegal, and Zambia.

5 The model could be calibrated to other data sources, and this will be a focus in future work.

6 As a comparison point, for the six PISA-D countries that also have learning poverty measures by the World Bank, the average learning poverty level is 67 percent. Across all low- and middle-income countries with learning poverty measures learning poverty is 53 percent and in low-income countries it is 89 percent, so the PISA-D countries are within reasonable levels for these income levels.

7 The model could be run with a shock to any grade cohort. Grade 3 was chosen as some learning has already occurred, and enough schooling remains to model long term consequences of the shock.

8 A similar assumption has been used in other modelling efforts (Cummiskey and Stern, 2020). Given that the majority of low-income countries have no announced distance learning plan, it is not unreasonable to assume that learning will effectively cease while children are out of school (Carvalho and Hares, 2020).

9 Treating grade 1 as a baseline, the average in-school grade 1 child in the counterfactual of no shock learns 36 points on the PISA-like scale per year, and in this shock scenario grade 10 children are 38 points behind the counterfactual of no shock.

10 The long term learning losses, in terms of years of learning lost, are larger for children in lower grades at the time of the shock because the losses have more years of schooling across which to accumulate, and smaller for children in higher grades. For example, alternative specifications show that an initial loss of one-third of a year for grade 1 students results in grade 10 learning that is 1.5 years lower, while initial loss of one-third of a year for grade 6 students results in grade 10 learning that is 0.5 years lower. An interactive data visualization tool is available for exploration of further scenarios: https://riseprogramme.org/tools/simulating-learning
